# Models of antenatal care to reduce and prevent preterm birth: a systematic review and meta-analysis

**DOI:** 10.1136/bmjopen-2015-009044

**Published:** 2016-01-12

**Authors:** Cristina Fernandez Turienzo, Jane Sandall, Janet L Peacock

**Affiliations:** 1 Division of Infection and Immunity, University College London, Hospital for Tropical Diseases, London, UK; 2 Division of Women's Health, Faculty of Life Sciences & Medicine, King's College London, Women's Health Academic Centre, St Thomas' Hospital,, London, UK; 3 Division of Health and Social Care Research, Department of Primary Care and Public Health Sciences, King's College London, London, UK

**Keywords:** OBSTETRICS, PUBLIC HEALTH

## Abstract

**Objective:**

To assess the effectiveness of models of antenatal care designed to prevent and reduce preterm birth (PTB) in pregnant women.

**Methods:**

We conducted a search of seven electronic databases and reference lists of retrieved studies to identify trials from inception up to July 2014 where pregnant women, regardless of risk factors for pregnancy complications, were randomly allocated to receive an alternative model of antenatal care or routine care. We pooled risks of PTB to determine the effect of alternative care models in all pregnant women. We also assessed secondary maternal and infant outcomes, women's satisfaction and economic outcomes.

**Results:**

15 trials involving 22 437 women were included. Pregnant women in alternative care models were less likely to experience PTB (risk ratio 0.84, 95% CI 0.74 to 0.96). The subgroup of women randomised to midwife-led continuity models of antenatal care were less likely to experience PTB (0.78, 0.66 to 0.91) but there was no significant difference between this group and women allocated to specialised care (0.92, 0.76 to 1.12) (interaction test for subgroup differences p=0.20). Overall low-risk women in alternative care models were less likely to have PTB (0.74, 0.59 to 0.93), but this effect was not significantly different from that in mixed-risk populations (0.91, 0.79 to 1.05) (subgroup p=0.13).

**Conclusions:**

Alternative models of antenatal care for all pregnant women are effective in reducing PTB compared with routine care, but no firm conclusions could be drawn regarding the relative benefits of the two models. Future research should evaluate the impact of antenatal care models which include more recent interventions and predictive tests, and which also offer continuity of care by midwives throughout pregnancy.

**PROSPERO registration number:**

CRD42014007116.

**Strengths and limitations of this study**

- This study is the first systematic review and meta-analysis to evaluate the efficacy and safety of existing models of antenatal care as a means of reducing preterm birth (PTB) rates in all pregnant women.
- Our study provides evidence from 15 randomised controlled trials with 22 437 participants. Comprehensive search strategies with no restrictions on publication date, country or language mean it is unlikely trials were missed.
- Compared to routine care, alternative models of antenatal care were associated with a significant reduction in the risk of PTB (16%). We performed subgroup analyses and sensitivity analyses, and assessed whether effects were different for low and mixed risk women and different models of antenatal care (midwife-led continuity of care and specialised antenatal care).
- Lack of high quality and substantial heterogeneity in some of the trials may have an influence on the power of this study.
- Lack of consistency in measuring and reporting women's experience and health economic evaluations can make them difficult to assess and report maternal satisfaction and economic costs.

## Introduction

Preterm birth (PTB) is the term used to define births that occur before 37 completed weeks of gestation.^1^ More than 1 in 10 babies worldwide are born prematurely every year, for an estimated 15 million PTBs, of which over one million die annually from complications of their prematurity.^2^ Many of the babies who survive face greater risks of significant health problems and disability throughout their lives (ie, learning disabilities, visual and hearing problems, chronic lung disease and other long-term diseases),^3^ which translate into significant increased costs to healthcare, the economy and the broader society.^4^

Despite numerous efforts to decrease its prevalence, improve clinical management and reduce neonatal morbidity and mortality, PTB rates continue to rise in most countries with reliable data.^5^ A wide variety of predisposing factors have been associated with PTB such as infections; social stress and intimate partner violence; non-Caucasian ethnic groups and other maternal factors (eg, young or advanced age; previous PTB; short inter-pregnancy intervals; nutritional deficiencies, cervical procedures; underlying medical conditions; smoking and alcohol consumption).^6^ This complex and multifactorial nature of PTB is likely responsible for single interventions not demonstrating a significant public health effect.^7^ However, there is a paucity of appropriately and efficaciously designed antenatal care packages for prematurity, and it remains a top research priority for PTB.^8^

We aimed to analyse, quantify and evaluate the effectiveness of existing or promising interventions delivered as an integrative package of antenatal care or models of care as a means of reducing PTB rates in all pregnant women, regardless of risk factors for pregnancy and birth complications. Our secondary objective was to assess their impact on antenatal hospitalisation, breastfeeding initiation, caesarean birth, induction of labour, instrumental vaginal delivery (forceps/vacuum), maternal satisfaction, spontaneous vaginal birth (as defined by trial authors), Apgar score ≤7 at minute five, admission to special care/neonatal intensive care unit (NICU), length of neonatal stay (mean length in days), low birthweight (<2500 g), fetal loss/neonatal death before and after 24 weeks, and overall fetal loss and neonatal deaths. Additionally, we aimed to perform subgroup analyses and sensitivity analyses of the main outcome (PTB) and to assess whether effects were different for low and mixed risk women and different models of antenatal care, and synthesise data on women's experiences and economic outcomes reported in the above trials.

## Methods

### Search strategy and selection criteria

**Figure 1:**
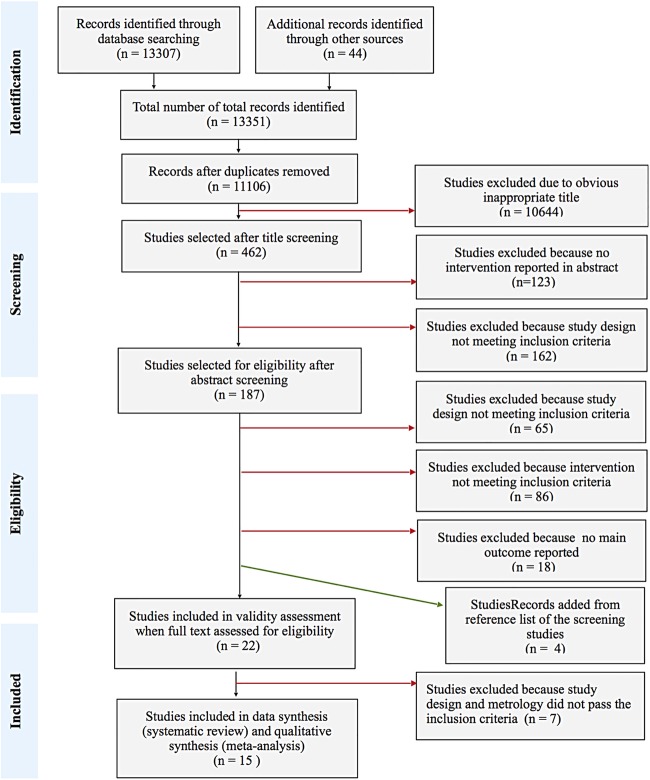
PRISMA Flow Diagram of Included Studies. PRISMA, Preferred Reporting Items for Systematic reviews and Meta-Analyses.

We conducted a systematic review of the effect of models of antenatal care on PTB according to Preferred Reporting Items for Systematic reviews and Meta-Analyses (PRISMA) guidelines^9^ (see figure 1). We comprehensively searched the following electronic databases, with no language, setting or time limits set, for published studies up to 14 June 2014: MEDLINE, EMBASE, CINAHL, CENTRAL, BNI, PsycINFO, and WEB of SCIENCE. Limitations to females and humans were applied to each database and highly sensitive search filters were used to identify reports of randomised controlled trials (RCTs).^10^ Search terms, keywords and strategies, which were reviewed by an information specialist in medicine, are detailed in online supplementary appendix 1. We also hand-searched bibliography and reference lists of the studies identified and other reviews to locate further studies and the Australian New Zealand Clinical Trials Registry and the US ClinicalTrials.gov register for unpublished and ongoing trials.

We included studies of pregnant women who were classified as being at low or high risk of pregnancy complications and/or PTB, regardless of their age, ethnicity, socioeconomic status or presence of comorbid conditions at enrolment. Studies using alternative models of antenatal care compared with standard care as a control were included. The intervention had to involve the organisation and provision of either comprehensive antenatal care or components of antenatal care delivered in the context of normal antenatal care. We included midwife-led continuity models of care, PTB prevention programmes, clinic-based specialised care and stand-alone interventions involving the provision of health or social care delivered in conjunction with standard antenatal care. No limitations were set regarding the professional who delivered the intervention. We excluded studies of stand-alone interventions or specific clinical and medical interventions targeting pregnant women, unless evaluated in the context of an integrated antenatal care package. Owing to existing reviews, studies of group prenatal care and packages of community care which did not include clinical care were excluded. Only studies that reported at least the main outcome measure of interest (PTB before 37 completed weeks’ gestation as the standard cut-off point) were included in the meta-analysis.

We selected studies in two stages. First, titles and abstracts were screened by one author (CFT) who excluded citations that were not related to RCTs or any model of antenatal care; second, the full text of potentially relevant studies was checked in a non-blinded, standardised manner, by a second reviewer (JS). The Cochrane Validity Inclusion Criteria was used to reassess the validity of the study design and the methodological details to determine whether the study should be included in the review^11^ (see online supplementary appendix 2). Final selections were based on consensus reached though discussion between the two reviewers.

Data extraction of the eligible studies was conducted using a predesigned template form. Two review authors (CFT and JS) independently extracted the data using the agreed form and any discrepancies were easily resolved though discussion. We used the Review Manager (RevMan) software V.5.3^12^ to double enter all the data. Thirty items of data were extracted from each study including details of settings, baseline characteristics of participants, details of experimental and control interventions, inclusion and exclusion criteria of the study participants, details of the risk assessment criteria used to identify women at low or high risk of PTB, number of total participants, loss to follow-up, and details regarding the definition of PTB as described by each study to validate the PTB outcome measurement, or if relevant, units of measurement. When information regarding any of the above was unclear, we contacted authors of the original studies to provide further details. For characteristics of included and excluded studies, see online supplementary appendices 3 and 5.

### Quality assessment

We used the criteria outlined in the *Cochrane Handbook of Systematic Review of Interventions*^13^ to make explicit judgements regarding whether or not the studies were at low or high risk of bias. The first reviewer (CFT) independently assessed and presented individual specific domains, including random sequence generation, allocation concealment, blinding, incomplete outcome data and selective reporting. Then, quality assessments were double-checked by a second reviewer (JS), and any discrepancies were resolved by discussion (see online supplementary appendix 6).

### Statistical analysis

The general meta-analytic approach was as follows. Outcomes from different trials were pooled where they were measured in the same way and random-effects pooled estimates were calculated throughout due to the nature and complexity of the interventions^14^ using RevMan.^12^ Dichotomous data are presented as pooled risk ratios (RR) with 95% CI and continuous data as summary mean difference (MD) with 95% CI.

We used the Cochrane Handbook^13^ as a guide to deal with the approximate analyses of a cluster trial^15–19^ included in the analysis. However, because an estimate of the intracluster correlation coefficient (ICC) was not provided for this study, a published ICC from another antenatal care trial was used^20^ alongside a sensitivity analysis to check the effect of varying the ICC was performed with to compare the two methods (see online supplementary appendix 7).

Statistical heterogeneity was assessed using τ^2^, I^2^ and χ^2^ statistics with its equivalent p value. Heterogeneity was regarded as substantial if I^2^ >30% and either if τ^2^ >0 or p<0.10. Potential sources of heterogeneity were investigated using subgroup analyses testing by fitting an interaction term. Subgroup analyses were stipulated a priori according to the review's main outcome, PTB and performed by type of intervention (midwife-led continuity models of care vs specialised antenatal care) and by level of risk status of the participants (low vs mixed risk). Funnel plots were used to examine reporting/publication bias where there were 10 or more studies for an outcome.

Study factors were used in sensitivity analyses to evaluate the impact of the methodological quality on the overall results. For the purpose of this review, studies were particularly considered to be of high quality if they used adequate methods of generating the allocation sequence and concealment and had an attrition rate <20%.

Maternal experience and economic costs of the antenatal care models were only reported narratively in due to a lack of consistency in measuring and assessing such outcomes.

## Results

Search strategies of the electronic databases and additional resources elicited a total of 13 351 citations, leaving 11 106 unique studies after duplicates were removed. Following title and abstract screening, the remaining 187 studies were selected for full-text assessment of eligibility. One hundred and sixty-five studies were excluded and another four studies were added from the reference and bibliography lists of the screened studies. Subsequently, of the 22 included studies assessed for validity, only 15 studies met the inclusion criteria for data analysis. The appendix lists include the validity assessments and reasons for exclusion.

We included a total of 15 RCTs^15–19^ ^21–48^ involving 22 437 women that were conducted in four countries in a wide variety of settings and health systems. The Zelen method was used in one trial,^27^ one study used multicentre cluster randomisation,^15–19^ and three other studies used multicentre individual randomisation.^24^ ^26^ ^27^ ^40^ ^41^ All of the included studies involved women classified as low-risk or mixed-risk for complications, and alternative models of antenatal care, which included either midwife-led continuity of care models or specialised antenatal care models.

Midwife-led continuity of antenatal care can be defined as the care where the midwife is the lead profesional in the planning, organisation and delivery of antenatal care given to a woman throughout her pregnancy. Some antenatal care may be provided in consultation with medical staff as appropriate. However, the composition, level of continuity and modus operandi of teams in midwife-led models varied among trials. Midwife-led continuity care models were compared to shared antenatal care models led by different healthcare professionals,^21^ ^22–23^ ^37^ ^38–39^ to medical-led models of care led by obstetricians,^29^ ^42–46^ and to various options of standard antenatal care, including rostered midwife-led, medical-led and shared care.^32–36^ ^47^ ^48^ Specialised care studies focused mainly on the value of specialised antenatal clinics for pregnant women identified at increased risk of PTB compared with standard antenatal clinic attendance. The studies compared the alternative care models with routine antenatal care provided by obstetricians, general practitioners (GPs), or both, in collaboration with midwives and nurses.^24–28^ ^30^ ^31^ A summary of the characteristics of included studies is presented in table 1A, B.

**Table 1:** Characteristics of included studies

Baseline characteristics									Summary of participants						
			Age of participants	Gestation at first visit			Socioeconomic status and/or education level*		Participants included in the study			Allocation of participants		Multiple pregnancies	Loss to follow-up
Study ID: major publication	Country	Years	Mean±SD or age groups (%)	Mean±SD	Ethnicity (%)	Marital status: married (%)	Trends	Other health behaviours (%)	Type	Classification	Number	Intervention group	Control group	Number (%)	Number (%)
Begley *et al*^21^	Ireland	2004–2007	30±4.9	NA	NA	58%	Higher	NA	Low risk	Low risk	1653	1101	552	NA	8 (0.5%)
Biro *et al*^22^	Australia	1996–1998	28±5.3	14±2.8	NA	70%	Lower	Smokers 35%	Low and high risk	Mixed risk	1000	502	498	25 (2.5%)	32 (3.2%)
Collaborative group 1993^24^	USA	1983–1986	NA	NA	Black: 44.2% Hispanic: 15.1% White: 39.7%	NA	Lower	NA	High risk	Mixed risk	2395	1200	1195	132 (10.7%)	NA
Goldenberg *et al*^25^	USA	1982–1986	<20: 36% 20–34: 60% ≥34 : 4.2%	NA	Black: 72% White: 27.7%	A minority	Lower	NA	High risk	Mixed risk	969	491	478	37 (7.7%)	NA
Hobel *et al*^15^	USA	1983–1988	26±5.6	19±7.1	Black: 8.2% Hispanic:75.5% White: 13.5% Asian: 4.4%	55%	Lower	NA	High risk	Mixed risk	2654	1174	880	19 (0.8%)	<10%
Iams and Johnson 26	USA	1983–1986	NA	NA	White and African American	NA	NA	NA	High risk	Mixed risk	370	182	188	NA	NA
Klerman *et al*^28^	USA	1994–1996	16–19: 29% 20–29: 61% ≥30: 11%	10±4.5	African- American:100%	7%	Lower	Smokers 21%	High risk	Mixed risk	619	318	301	NA	NA
MacVicar *et al*^29^	UK	1989–1991	25±4.5	NA	NA	NA	NA	Smokers 28%	Low risk	Low risk	3510	2304	1206	NA	NA
Main *et al*^30^	USA	1983–1986	24±5.3	12.2±3.4	Black: 100%	NA	Lower	NA	High risk	Mixed risk	943	198	178	0 (0%)	NA
McLachlan *et al*^32^	Australia	2007–2010	31±4.7	16.3±2.8	NA	95%	Higher	Smokers 3.5%	Low risk	Low risk	2314	1156	1158	NA	7 (0.3%)
Rowley *et al*^37^	Australia	1991–1992	26±2.3	NA	White: >90%	>50%	Higher	Smokers <50%	Low and high risk	Mixed risk	814	405	409	13 (1.6%)	NA
Tracy *et al*^38^	Australia	2008–2011	31±4.9	NA	NA	NA	Higher	NA	Low and high risk	Mixed risk	1748	871	877	0 (0%)	45 (2.5%)
Tucker *et al*^40^	UK	1993–1994	25±0.3	12.2±0.2	NA	34%	NA	Smokers 30%	Low risk	Low risk	1765	878	877	NA	91 (5.5%)
Turnbull *et al*^42^	UK	1993–1994	26±5.0	NA	NA	54%	Lower	Smokers 38%	Low risk	Low risk	1299	648	651	NA	21 (1.6%)
Waldenström^47^	Australia	1996–1997	28±5.2	12.2±4.2	NA	89%	Higher	Smokers 37%	Low risk	Low risk	1000	495	505	6 (0.6%)	20 (2%)



Summary of interventions							
		Content of intervention					
Study ID: major publication	Type	Antenatal care. Frequency of visits	Cervical examinations	Health Education	Psychosocial support and/or others	Delivery	Setting
Begley *et al*^21^	Midwife-led continuity model of care	Yes. Routine visits or more depending on complications				Midwives (GPs if desired)	Hospital based and out-reach clinics
Biro *et al*^22^	Midwife-led continuity model of care	Yes. Routine visits or more depending on women's risk status				Midwives	Hospital-based clinic
Collaborative Group 1993^24^	Specialised care	Yes. Weekly visits from 20 to 24 weeks’ until delivery	Yes	Yes		Specialist nurses and medical staff	Community-based clinic
Goldenberg *et al*^25^	Specialised care	Yes. Weekly visits from 22 weeks until delivery	Yes	Yes.		Specially trained nurses	Community-based clinic
Hobel *et al*^15^	Specialised care	Yes. Biweekly visits until delivery		Yes.	Yes. Psychosocial and nutritional counselling. Secondary interventions (bed rest, psychosocial support, oral progestin, or a placebo)	Nurses, health educators, nutritionists	Community-based clinic
Iams and Johnson^26^	Specialised care.	Yes. Weekly visits from 20–36 weeks	Yes	Yes		Not clearly stated	Specialised clinic
Klerman *et al*^28^	Specialised care.	Yes. Biweekly visits until 36 weeks’ gestation, then weekly		Yes	Yes. Group sessions: pregnancy, peer social support, healthy behaviours.	Nurses and trained community experienced workers	Community-based clinic
MacVicar *et al*^29^	Midwife-led continuity model of care	Yes. Routine visits (one to three visits)				Midwives	Hospital-based clinic
Main *et al*^30^	Specialised care,	Yes. Weekly or biweekly visits from 22 weeks to birth	Yes	Yes		Physicians and specialists nurses	Hospital based specialised clinic
McLachlan *et al*^32^	Midwife-led continuity model of care	Yes. Routine visits (one to three visits)				Caseload midwives	Community-based clinic.
Rowley *et al*^37^	Midwife-led continuity model of care	Yes. Routine visits (one to three) or more depending on risk status				Midwives	Hospital-based clinic
Tracy *et al*^38^	Midwife-led continuity model of care	Yes. Routine visits or more depending on risk status				Caseload midwives	Community-based clinic
Tucker *et al*^40^	Specialised care	Yes. Visits arranged according to detailed care plans				GP and community midwives	Community-based clinic
Turnbull *et al*^42^	Midwife-led continuity model of care	Yes. Routine visits or more depending on arising complications				Caseload midwives	Community-based clinic
Waldenström *et al*^47^	Midwife-led continuity model of care	Yes. Routine visits or more depending on arising complications				Midwives	Hospital-based clinic

*Trend toward lower or higher socio-economic status and education levels across study participants.

GP, general practitioners; NA, not applicable or not reported.

A few trials excluded women who were at more than 24 weeks’ gestation, more than 30 weeks’ gestation,^25^ or more than 32–34 weeks’ gestation.^24^ Women with a multiple pregnancy or a planned elective caesarean section were excluded from two trials.^30^ ^31^ ^38^ ^39^ Some studies excluded women with substance abuse problems,^28^ ^37^ or attempted suicide during pregnancy.^15–19^ Other studies excluded women with complex general medical conditions (ie, epilepsy, diabetes, cancer, HIV/AIDS or cardiac disease);^15–19^ ^28^ or women with previous small-for-gestational-age babies, stillbirth or neonatal death.^30^ ^31^ Online supplementary appendices 3, 4 and 8 include detailed characteristics of included studies, reported outcomes of interest and a brief description of the risk assessment criteria used to classify the women's risk status. All of the 15 included studies used the WHO definition criteria when stating the main outcome of this review (PTB).^1^

### Effects of alternative models of antenatal care versus routine care for all pregnant women

**Figure 2:**
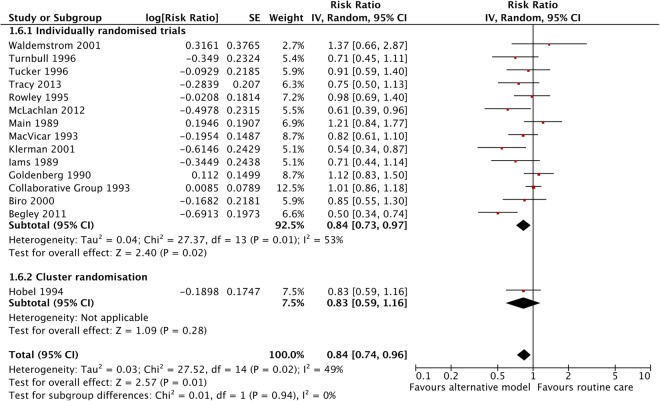
Forest plot comparing preterm birth (<37 weeks) between pregnant women receiving alternative models of antenatal care and those receiving routine care—adjusted for cluster design effect (ICC 0.002). ICC, intracluster correlation coefficient.

Compared to women in routine care groups, pregnant women in alternative models of antenatal care were, on average, less likely to experience PTB <37 weeks (RR 0.84, 95% CI 0.74 to 0.96) (figure 2). In addition, these women are, on average, less likely to experience a caesarean birth (RR 0.92, 95% CI 0.85 to 1.00) and induction of labour (RR 0.90, 95% CI 0.81 to 0.99); and more likely to experience spontaneous vaginal birth (as defined by the trial authors) (RR 1.05, 95% CI 1.01 to 1.10) (see online supplementary figures 9.3, 9.4 and 9.6 in appendix 9). There were no statistically significant differences between alternatives care models and routine care groups in any of the remaining outcomes (see online supplementary figures 9.1, 9.2, 9.10–9.14, 9.17 and 9.18 in appendix 9). Table 2 presents a summary of the meta-analysis on maternal and neonatal outcomes of interest.

**Table 2:** Summary of data analysis on outcomes of interest

Outcome	Studies (N)	Participants	Pooled RR (95% CI)	I^2^ (%)	p Value
Primary outcomes					
Antenatal hospitalisation	4	5187	1.02 (0.93 to 1.11)	15	0.72
Breastfeeding initiation	3	5067	1.01 (0.95 to 1.08)	56	0.75
Caesarean birth	11	15 919	0.92 (0.85 to 1.00)	2	0.04
Induction of labour	9	14 924	0.90 (0.81 to 0.99)	59	0.03
Instrumental vaginal delivery (forceps/vacuum)	9	14 924	0.93 (0.86 to 1.01)	0	0.08
Preterm birth (<37 weeks)—adjusted for cluster design effect (ICC 0.002)	15		0.84 (0.74 to 0.96)	49	0.01
Individualised randomised trials	14		0.84 (0.73 to 0.97)		
Cluster randomisation	1		0.83 (0.59 to 1.16)		
Preterm birth (<37 weeks)—sensitivity analysis (ICC 0.0041)	15		0.83 (0.72 to 0.95)	51	0.007
Individualised randomised trials	14		0.84 (0.73 to 0.97)		
Cluster randomisation	1		0.69 (0.47 to 1.02)		
Preterm birth (<37 weeks)—all studies, unadjusted data	15	22 437	0.86 (0.76 to 0.97)	42	0.01
Individualised randomised trials	14	19 783	0.86 (0.75 to 0.98)		
Cluster randomisation	1	2654	0.81 (0.62 to 1.06)		
Spontaneous vaginal birth (as defined by trial authors)	9	14 924	1.05 (1.01 to 1.10)	65	0.01
Secondary outcomes					
5-min Apgar score ≤7	9	10 779	0.86 (0.68 to 1.09)	0	0.21
Admission to special care unit/NICU	11	15 225	0.94 (0.84 to 1.05)	15	0.25
Fetal loss/ neonatal death before 24 weeks	9	14 968	0.81 (0.65 to 1.02)	0	0.07
Fetal loss/ neonatal death equal to/after 24 weeks	8	13 294	0.97 (0.60 to 1.58)	0	0.91
Low birthweight (<2500 g)—adjusted for cluster design effect (ICC 0.0003)	10		0.83 (0.61 to 1.13)	85	0.23
Individualised randomised trials	9		0.77 (0.55 to 1.09)		
Cluster randomisation	1		1.38 (1.00 to 1.92)		
Low birthweight (<2500 g)—sensitivity analysis (ICC 0.0016)	10		0.82 (0.60 to 1.12)	78	0.22
Individualised randomised trials	9		0.77 (0.55 to 1.09)		
Cluster randomisation	1		1.36 (0.92 to 2.00)		
Low birthweight (<2500 g)—all studies, unadjusted data	10	17 992	0.98 (0.89 to 1.09)	0	0.76
Individualised randomised trials	9	15 338	0.99 (0.89 to 1.11)		
Cluster randomisation	1	2654	0.91 (0.67 to 1.25)		
Mean length of neonatal hospital stay (days)			**MD (95% CI)**		
	3	2027	−2.11 (−4.64 to 0.41)	69	0.10

ICC, intracluster correlation coefficient; MD, mean difference; NICU, neonatal intensive care unit; RR, risk ratios.

**Figure 3:**
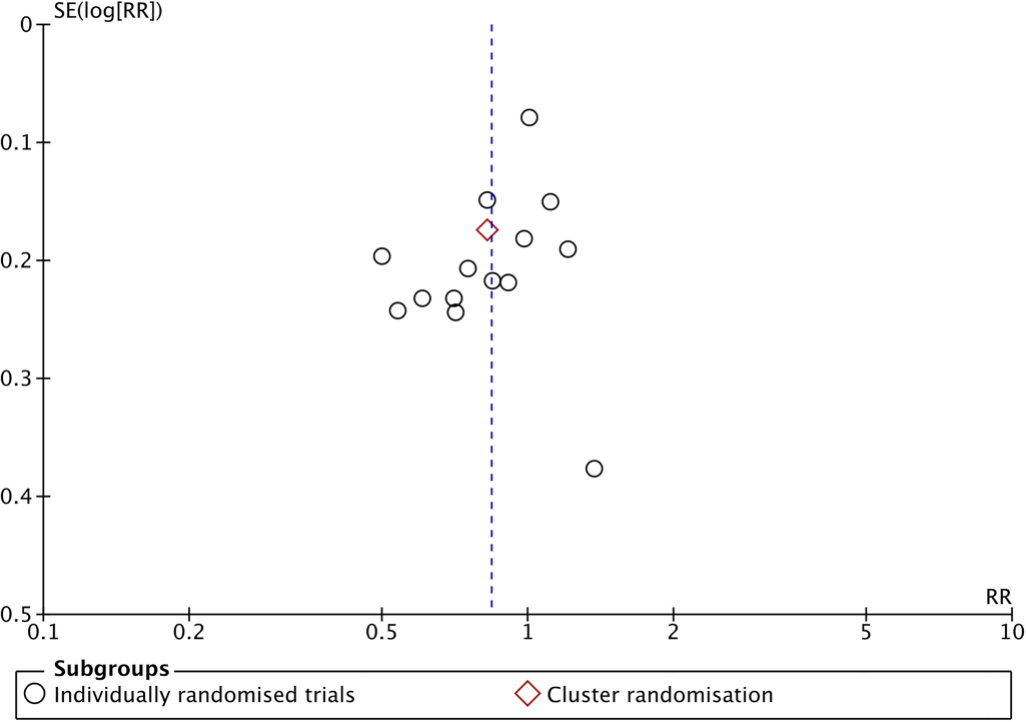
Funnel plot comparing preterm birth (<37 weeks) between pregnant women receiving alternative models of antenatal care and those receiving routine care—data adjusted for cluster design effect (ICC 0.002). ICC, intracluster correlation coefficient; RR, risk ratios.

There was substantial heterogeneity in many of the analyses. The I^2^ value was greater than 50% for six outcomes—breastfeeding initiation, induction of labour, spontaneous vaginal birth, low birthweight and mean length of neonatal hospital stay—and greater than 30% for PTB. However, little or non-significant heterogeneity was observed between studies in the remaining outcomes. There was slight asymmetry in the funnel plot for PTB (figure 3) due to a small trial with small number of events and large treatment effects.^47^ ^48^ Other funnel plots were mostly symmetrical, providing little overall evidence for publication bias (see online supplementary figures 9.19, 9.21 and 9.22 in appendix 9).

### Subgroup analysis of effects of different antenatal care models and mixed risk and low-risk populations of women on PTB outcome

**Figure 4:**
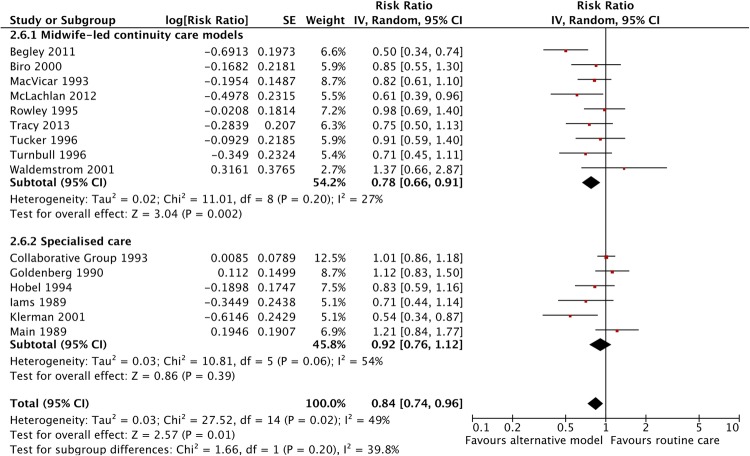
Forest plot comparing preterm birth (<37 weeks) outcome variation between midwife-led and specialised care for alternative models of antenatal care versus routine care.

**Figure 5:**
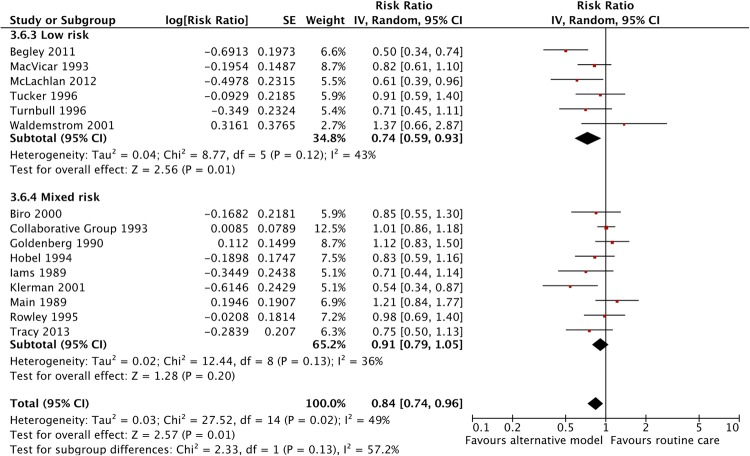
Forest plot comparing preterm birth (<37 weeks) outcome variation between pregnant women at low and mixed risk of complications for alternative models of antenatal care versus routine care.

There was a significant effect of midwife-led continuity care models on reducing PTB in the intervention group (RR 0.78, 95% CI 0.66 to 0.91), while specialised care models were not significant (RR 0.92, 95% CI 0.76 to 1.12) However, the interaction test shows that these two relative risks are not significantly different from each other (p=0.20) (figure 4). Similarly, there was no evidence that the effect in low-risk women while significant on its own (RR 0.74 95% CI 0.59 to 0.93), differed significantly (p=0.13) from that in mixed risk women (RR 0.91 95% CI 0.79 to 1.05) (figure 5).

### Sensitivity analysis

A sensitivity analysis was performed for the PTB outcome by using the upper 95% CI for the ICC (0.0041). Thus, the weight of the study was reduced from 7.6% to 6.7%. However, this adjustment made very little difference, and the overall results remained significant with substantial heterogeneity (RR 0.83, 95% CI 0.72 to 0.95) (see online supplementary figure 9.8 in appendix 9). Similarly, the sensitivity analysis performed for the low birthweight outcome by using the upper 95% CI for the ICC (0.0016) reduced the weight of the study from 11% to 10.5%, but the overall intervention effect was not significant (RR 0.82, 95% CI 0.60 to 1.12), and subgroup differences remained statistically significant in the presence of substantial heterogeneity (see online supplementary figure 9.15 in appendix 9).

Overall, the sensitivity analysis for PTB including only high-quality studies^21–23^ ^32–36^ ^38–46^ did not alter the results for the main outcome, which remained consistent with the overall findings. Studies rated at high risk of bias were excluded in the sensitivity analysis in order to assess for any substantive difference to the overall findings (see online supplementary fig 9.23 in appendix 9).

### Maternal experience and satisfaction and economic analysis

The lack of consistency in measuring and reporting women's experience and satisfaction and health economic evaluations, makes the presentation of a narrative synthesis of such data much more feasible.^13^ Seven studies reported maternal satisfaction with diverse elements of the antenatal experience.^23^ ^28^ ^29^ ^37^ ^40–48^ Considering the ambiguity associated with the concept of satisfaction, it was expected to find inconsistencies in the instruments, scales, timing and outcomes used to ‘measure’ women's experiences with antenatal care across studies. Maternal experience and satisfaction was considered with information, explanation, advice, duration of antenatal visits and participation in decision-making. One study^28^ assessed also perceptions of antenatal risks, including PTB risk, reported behavioural changes and degree of control over events in the participants’ lives (perceived mastery) using three—and four—point scales. The overall satisfaction indicators and rating scores, which directly related to both provider attitude and/or women, are presented as tabulated results for easy understanding and convenience in online supplementary appendix 10. In brief, most of the included studies showed higher satisfaction levels with antenatal care in both alternative models (midwife-led continuity care and specialised care) when compared to routine antenatal care.

Six studies included in this review provided an economic analysis, including various items and measures in the final cost estimation; however, one of the studies included only inpatient maternity care, excluding antenatal or postnatal care cost estimation.^37^ In summary, only five studies presented maternity cost data of interest, using diverse methods for economic evaluation.^15–19^ ^30^ ^31^ ^38–46^ The analysis of two included studies suggested a higher cost of antenatal care programmes designed for high-risk women,^15–19^ ^30^ ^31^ and the analysis of three included studies suggested a cost-saving effect in midwifery-led care compared to standard shared care.^38–46^ Overall, there was a lack of consistency among the available studies in estimating the costs of antenatal care; however, there appears to be a trend appears towards the cost-saving effects of midwife-led continuity of antenatal care compared with specialised care. Economic costs of the antenatal care models in included studies are reported narratively in detail in online supplementary appendix 11.

## Discussion

The results of our main review comparison indicated that the risk of PTB <37 weeks in pregnant women who received alternative antenatal care models was reduced by 16%, compared with women who received routine care. In addition alternative antenatal care models were associated with reductions in risk of induction of labour (by 10%) and caesarean birth (by 8%) and with increased likelihood of spontaneous vaginal birth (by 5%). These results were robust to various sensitivity analyses. There was no evidence that any alternative model of antenatal care was associated with an increased likelihood of adverse outcomes for women or their infants. Instrumental vaginal delivery and fetal loss before 24 weeks were less likely in women receiving alternative antenatal care models but were not statistically significant.

There were considerable variations in the interventions and participants across studies and so interpreting the findings of these main comparisons was not straightforward. Trends in subgroup analyses suggested that the type of intervention and women's risk status might influence the effectiveness of the intervention, but the results were not statistically significant. Compared with studies of midwife-led continuity care, most specialised clinic studies provided little data on the outcomes of interest, with the exception of PTB. In fact, the overall beneficial effects of PTB, spontaneous vaginal birth, induction of labour, and caesarean birth, were obtained from an analysis of midwife-led continuity of care studies. The observation that pregnant women allocated to midwife-led continuity care models were less likely to experience PTB compared to women allocated to specialised clinic care is potentially important given the global interest in preventing PTB^4^ ^49^ but this observed benefit was not statistically different from that observed for specialised care. Hence though while no firm conclusions can be drawn, the issue remains important due to the growing focus on specialised clinics to deal with the increasing complexity of managing women at high risk despite the paucity of evidence to support their benefit.^50^ It is possible that existing clinical interventions aimed at the prevention of PTB might depend on fast access to care, which is potentially influenced by midwifery continuity of care.^51^ This would be consistent with the observation that low-risk and mixed-risk women in midwife-led care models have a lower risk of PTB.

The observed results might also be partially explained by the varying type, content or delivery of the intervention. All studies of specialised PTB clinics included weekly or biweekly antenatal visits throughout the pregnancy and included detailed education regarding preterm labour onset signs and symptoms. Two studies included psychosocial support and nutritional counselling; however, programmes that offer additional support have not been shown to prevent PTB.^52^ Four other studies repeated digital cervical assessments, although there has been no evidence of its effectiveness as a screening test for PTB risk in average-risk pregnancies.^53^ Importantly, the studies of specialised care were conducted in the late 1980s and 1990s, before the development of new clinical interventions to prevent PTB such as progesterone or use of cerclage, and before promising screening tests to predict PTB.^54^ In the UK and the USA, recent reviews have advocated promising tests that predict the risk of PTB, including the fetal fibronectin test (FFT),^55^ combined approaches using FFT and transvaginal ultrasound scan for cervical length in symptomatic women,^56^ as well as the FFT, absence of fetal breathing movements, and cervical length within 48 h and within 7 days of testing.^57^

Interventions in the majority of specialised care studies were provided by specially trained staff, but little information was provided regarding adherence or safeguard checks to ensure programme differentiation in most of the studies. Late participant registration (middle and end of second trimester or later) in half of the studies might also have reduced the effectiveness of the interventions since longer exposure to specialised care is likely to be beneficial. The issue that all specialised care studies were conducted in the USA may potentially affect generalisability of these results. The USA has a health system wherein midwives were not be able to provide midwife-led continuity of care. Antenatal care in the USA is generally provided through care led by obstetricians, GPs, or both or shared among diverse health professionals.^58^ Thus, specially trained nurses were the main care providers in only three studies, and the level of continuity of care was unclear. The extent to which the reduced likelihood of PTB might be attributed to the continuity of antenatal care models or the degree and quality of relationships between women and their healthcare providers will require further research. In addition, the role of risk-scoring systems in the prevention of PTB is currently unknown.^59^ While the Creasy risk-scoring tool was commonly used to define women at low or high risk of PTB, score systems differed across trials, and the information regarding the cut-off used was not consistently reported. This might have affected, for example, the enrolment of women with relatively low-risk scores, potentially leading to the underenrolment of the women who had the most to benefit from the intervention.

Owing to the nature of the complex interventions, it was recognised that clinical heterogeneity was sufficient to expect that the underlying effects varied among studies. In fact, except for midwife-led studies, there was substantial statistical heterogeneity within each subgroup analysed. It is possible that women's psychosocial, structural, and sociodemographic characteristics, defined in terms of risk characteristics (eg, maternal behaviours, education, infections, stress or ethnic differences in genetics) might have had a confounding influence on PTBs.^60^ ^61^ Midwifery continuity models of care have been particularly valued by socially disadvantaged women with difficult access to health services,^62^ ^63^ and their experience of more empathic care, agency and control^64^ may have an impact on PTB outcomes. Worldwide, and particularly in the UK, there is an increasing interest in ensuring that specialised antenatal clinics offer midwifery continuity of care.^65^

In this review, there were limitations in the way maternal satisfaction and economic costs were assessed and reported in the eight studies examined. However, of the specialist care studies, only two reported economic aspects and only one reported maternal satisfaction. The level of satisfaction with several aspects of antenatal care appears to be higher in specialised care and midwife-led continuity models than in routine care. Despite a lack of consistency in estimating maternity care costs among the included trials, the results of various economic evaluation methods generally suggest a trend towards a cost-saving effect in midwife-led continuity care models and a higher cost of specialised care compared to routine care.

### Quality of the evidence

The strengths of this review include the use of comprehensive search strategies and the rigorous methods used for the data synthesis. Despite the lack of restrictions regarding publication date, country or language, all of the included studies were written in English, published between 1989 and 2013, and conducted in high-income countries in different cultural and healthcare system settings.

**Figure 6:**
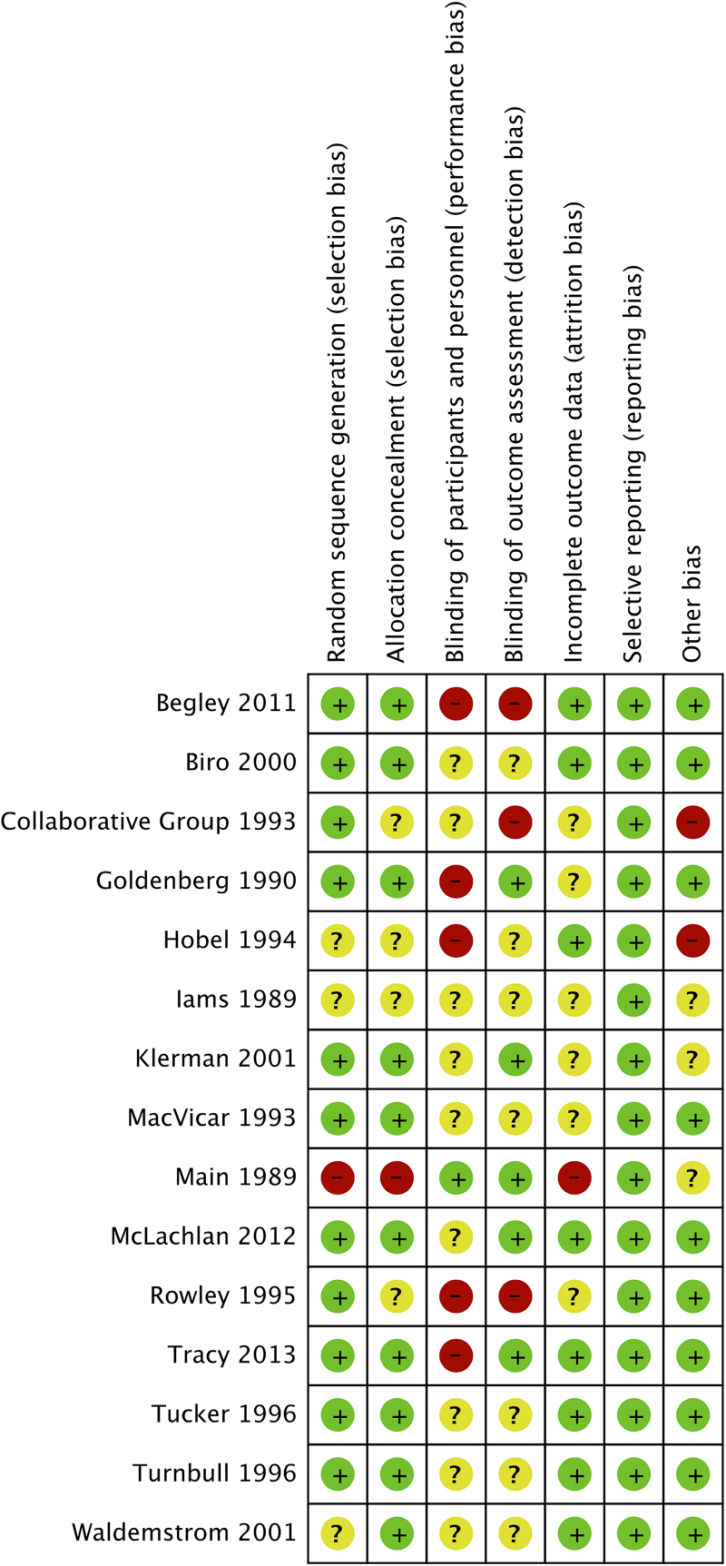
Risk of bias summary showing review author's judgements about each risk of bias domain in included studies. Randomised clinical trials are listed alphabetically by author name.

The quality of the evidence in the 15 included studies was mixed, and this could be linked to methodological insufficiencies and substantial heterogeneity in some studies. Figure 6 summarises the assessment of risk of bias for individual trials. Compared with studies of midwife-led continuity of care, studies of specialised care were of lower quality. Most of these studies reported gestational age at delivery, preterm labour or PTB with little information regarding other outcomes of interest; statistical power may not have been sufficient to detect significant differences between subgroups. Only one study used a quasi-randomised method, and the methods used to conceal allocation were at unclear or high risk of bias in four other studies in which the women were randomised. The blinding methodology was not clearly reported in half of the included studies, and some of the included studies were at high risk of bias in blinding of outcome. Findings of the cluster trial were not adjusted for cluster design effect, and possible design effects might have been taken into account for PTB and low birthweight outcomes. Two midwife-led continuity of care studies and four specialised care studies reported no or insufficient information on incomplete outcome data, possibly affecting the quality of the evidence. Even small losses might sometimes be significant, and the women who were most vulnerable to adverse outcomes might have been over-represented among those who were not followed up. Steps were taken to minimise potential biases in the reviewing process, including the two authors independently assessing study eligibility, as well as data extraction and meta-analysis.

## Authors’ conclusions

### Implications for practice and research

Our findings suggest that overall, alternative models of antenatal care for all pregnant women are effective in reducing PTB compared with routine care without any evidence of adverse effects. No firm conclusions could be drawn regarding the relative benefits of the two models, midwife-led continuity of care and specialised care. However, because a few studies excluded women with substantial maternal disease, substance misuse or suicide attempts during pregnancy, caution is needed when applying these findings to pregnant women with the above medical, mental and/or obstetric complications.

Substantial progress is still required in the research and implementation of the evidence.^66^ A better understanding of the multicausal and complex nature of PTB might help to develop an effective framework for trials of more recent interventions in reducing the rate of PTB. Future research will require an exploration of whether the midwife-led continuity model of antenatal care or the degree and quality of relationship between the woman and the care provider might be responsible for the significant impact on PTB. In addition, more research on effective risk-screening tools to predict preterm labour and a data set of core outcome measures to be collected^67^ would be useful for making trial comparisons and for further reviews of similar studies. Future studies should consider treating gestational age as a continuum rather than a dichotomy since this better reflects the biological processes which do not change markedly at 37 weeks. Further, the analysis of gestational age as a continuous variable provides more statistical power and allows finer differences to be detected.^68^

In addition, studies of complex interventions such as antenatal care models should include standardised reporting of the intervention^69^ and implementation fidelity.^70^ Further research will be required to develop reliable and valid methods of assessing women's satisfaction and well-being, as well as standard approaches to estimate relative antenatal costs and benefits to women, families, societies, and the health systems.
